# Pericardial Tamponade in a Patient with Inactive Ulcerative Colitis

**DOI:** 10.1155/2010/352417

**Published:** 2010-02-28

**Authors:** Ali Rezaie, Karen Wong, Gabor Gyenes

**Affiliations:** ^1^Faculty of Medicine, University of Alberta, #409 11135 83 Ave Edmonton, Alberta, Edmonton, Canada T6G 2C6; ^2^Department of Gastroenterology, Faculty of Medicine, University of Alberta, Edmonton, Canada T6G 2X8; ^3^Department of Cardiology, Faculty of Medicine, University of Alberta, Edmonton, Canada T6G 2B7

## Abstract

Cardiac tamponade is a rare extraintestinal manifestation of ulcerative colitis. We present a case report of tamponade occurring four years after curative proctocolectomy and in the absence of any medical therapy for ulcerative colitis. Options for medical management of this condition are also discussed.

## 1. Introduction

Cardiac tamponade is a rare extraintestinal manifestation of ulcerative colitis (UC) with only nine reported cases in the literature. However, association of UC and tamponade has been called into question given the difficulty of complete exclusion of other causes, as well as concomitant use of sulphasalazine/5-aminosalicylic acid (5-ASA) that can adversely affect the pericardium [1].

We present a case which broadens the understanding of UC-associated pericarditis and tamponade by underlining that this condition may develop in the absence of concomitant 5-ASAs or sulphasalazine treatment and even several years after curative total colectomy. In addition, we emphasize that quick tapering of steroids may lead to recurrence of the disease, and colchicine may be beneficial in maintenance of remission.

## 2. Case Report

A 35-year-old male presented in May 2008 with low-grade fever and left upper quadrant abdominal pain. His past medical history comprised of UC for seven years, for which he was on no medication after a curative proctocolectomy with end-ileostomy were carried out in 2004. He also had postoperative neurogenic bladder and recurrent urinary tract infections (UTIs), asthma, and three-year history of self-limiting pleuritic chest pains. His medications included budesonide/formoterol inhaler, pantoprazole, vitamin D, and calcium.

Physical examination including vital signs and jugular venous pressure was within normal limits. Following a chest X-ray, CT scan of abdomen, and ECG that were all reported to be normal, the patient was admitted for observation and treatment with ciprofloxacin for UTI (Klebsiella pneumoniae and Escherichia coli).

On the fourth day of admission, he developed progressive left-sided pleuritic chest pain radiating to his back and shortness of breath. Physical examination revealed normal temperature, tachypnea, tachycardia, and hypotension. Jugular veins were engorged up to the angle of the jaw. No pericardial rub or murmur was appreciated. Lungs were clear to auscultation and abdomen was soft and nontender. No oral lesions, rash, arthritis, or peripheral edema were noted. 

**Figure 1:**
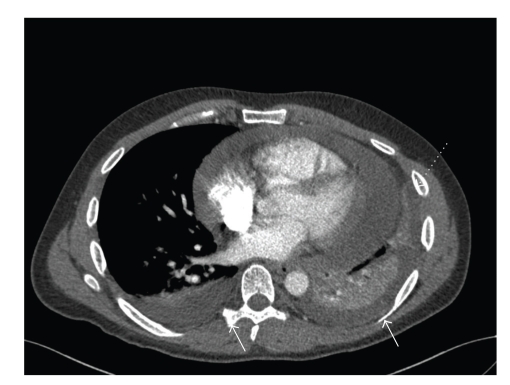
CT scan showing pericardial (dashed arrow) and bilateral pleural effusion (solid arrows).

Arterial blood gas showed PaO_2_ of 48, PCO_2 _ of 41 mmHg, and saturation of 84% on room air. Electrocardiogram showed ST elevation and PR depressions in all leads. CT angiography of chest did not show any pulmonary embolism but revealed moderate-size pericardial effusion and bilateral pleural effusions (Figure 1). Urgent transthoracic echocardiogram was carried out and showed cardiac tamponade. Subsequently, an urgent pericardial tap was performed and a total of 600 cc of clear fluid was drained over the next 3 days (Tables 1 and 2).

Patient was started on prednisone 70 mg daily and colchicine 0.6 mg twice daily, and pericardial drain was removed four days later. His symptoms completely resolved over the next 48 hours and he was discharged with a tapering schedule to reduce the dose of prednisone to 20 mg in two weeks. After two weeks, patient represented with pleuritic chest pain and recurrent ECG changes suggestive of pericarditis. Echocardiogram and chest X-ray did not reveal pericardial or pleural effusion. Prednisone dosage was increased to 50 mg daily and was tapered over a 3-month period while colchicine was continued at the same dose. As of twelve months of follow-up he has not experienced any clinical or ECG finding suggestive of recurrent pleuro-pericardial involvement. Of note that throughout the course of the disease the patient did not have any symptoms of active inflammatory bowel disease.

## 3. Discussion

Pericarditis is a known but rare complication of UC [2]. Recurrent and chronic pericarditis has also been reported [1]. With a much lower incidence, inflammatory bowel disease has been associated with pericardial tamponade (1 and 10 cases in Crohn's disease and UC, resp.) [1, 3]. 

As UC-associated pericardial effusion is a diagnosis of exclusion, previous case reports, particularly older reports, are scrutinized for lack of laboratory and microbiologic evidence to rule out secondary causes of pericardial effusion. In this case, extensive viral, bacterial, mycobacterial, and fungal studies were performed with negative results. In addition, connective tissue diseases, uremia, hypothyroidism, and malignant causes were excluded (Tables 1 and 2).

5-ASA and sulphasalazine have been associated with myopericardial involvement [4]; moreover, sulphasalazine can cause drug-induced lupus with a range of manifestations including pericardial involvement [5]. This patient has not received any of these compounds for more than four years making these drugs an unlikely cause for his condition.

Patient had not been started on any new medications except ciprofloxacin for UTI which to our knowledge is not associated with pericardial involvement.

Postoperative UC-associated tamponade has been reported in three cases following subtotal colectomy [3, 6, 7]. Occurrence of tamponade four years after total colectomy signifies that extraintestinal manifestation of UC can still occur despite inactive UC.

Lack of significant pericardial fluid on the CT scan four days prior to tamponade provided us with an opportunity to elaborate the acuteness of UC-induced tamponade.

Recurrence of pericardial disease after rapid tapering of the prednisone dose has been reported previously in UC patients [1]. Similar phenomenon occurred in this case; however, slow tapering of prednisone over three months similar to standard steroid therapy for UC flare-ups prevented the recurrence for the second time.

Despite the promising results of the COPE trial [8] on beneficial effects of colchicine in reduction of pericarditis recurrence, effect of colchicine on UC-associated pericardial disease has not been evaluated. Given the deleterious effects of NSAIDs on ulcerative colitis [9], colchicine was started in this patient. Lack of further symptoms after slow tapering and discontinuation of steroids might suggest a positive role for colchicine in maintaining the prednisone-induced remission in UC-induced pericarditis.

In conclusion, cardiac tamponade should be considered as a potential life-threatening extraintestinal manifestation of UC and be treated accordingly. It should be noted that currently a causal relationship between IBD and cardiac tamponade is not established.

## Figures and Tables

**Table 1:** Relevant blood and serologic findings.

Data	Laboratory results	Normal range
Blood and serologic findings		
Hemoglobin, g/L	129	135–175
WBC, 10^9^/L	7.1	4–11
Platelets, 10^9^/L	163	140–450
Total protein, g/L	62	64–84
Creatinine, *μ*mol/L	70	45–125
Urea, mmol/L	2.5	2.5–8.0
Creatinine kinase, unit/L	215	<250
Troponin, *μ*g/L	<0.10	<0.15
ESR, mm/hour	77	0–15
CRP, mg/L	35.6	<8.0
Lactate dehydrogenase, U/L	135	100–225
TSH, mU/L	0.73	0.2–4.0
ANA	Negative	Negative
Anti-ds DNA, %	2	0–15
Rheumatoid factor, kU/L	<9	<20
C3, g/L	1.76	0.8–2.0
C4, g/L	0.27	0.18–0.36
HIV serology	Negative	Negative

**Table 2:** Laboratory data on pericardial fluid.

Pericardial fluid analysis	
WBC, 10^6^/L	2000
Neutrophils, %	74
Lymphocytes, %	8
Monocytes, %	16
Eosinophils,%	2
Total protein, g/L	43
Lactate dehydrogenase, U/L	581
Glucose, mmol/L	4.3
Viral/bacterial/AFB culture	Negative
Shell vial culture for CMV	Negative
Enterovirus RNA	Negative
Cytology	Negative
